# Actinomycetes: A Source of Lignocellulolytic Enzymes

**DOI:** 10.1155/2015/279381

**Published:** 2015-12-17

**Authors:** Anita Saini, Neeraj K. Aggarwal, Anuja Sharma, Anita Yadav

**Affiliations:** ^1^Department of Microbiology, Kurukshetra University, Kurukshetra, Haryana 136119, India; ^2^Department of Biotechnology, Kurukshetra University, Kurukshetra, Haryana 136119, India

## Abstract

Lignocellulose is the most abundant biomass on earth. Agricultural, forest, and agroindustrial activities generate tons of lignocellulosic wastes annually, which present readily procurable, economically affordable, and renewable feedstock for various lignocelluloses based applications. Lignocelluloses are the focus of present decade researchers globally, in an attempt to develop technologies based on natural biomass for reducing dependence on expensive and exhaustible substrates. Lignocellulolytic enzymes, that is, cellulases, hemicellulases, and lignolytic enzymes, play very important role in the processing of lignocelluloses which is prerequisite for their utilization in various processes. These enzymes are obtained from microorganisms distributed in both prokaryotic and eukaryotic domains including bacteria, fungi, and actinomycetes. Actinomycetes are an attractive microbial group for production of lignocellulose degrading enzymes. Various studies have evaluated the lignocellulose degrading ability of actinomycetes, which can be potentially implemented in the production of different value added products. This paper is an overview of the diversity of cellulolytic, hemicellulolytic, and lignolytic actinomycetes along with brief discussion of their hydrolytic enzyme systems involved in biomass modification.

## 1. Introduction

Actinomycetes, a separate taxonomic group within domain bacteria, are members of the order Actinomycetales [1]. They are Gram positive bacteria, primarily aerobic and spore formers, with high G+C content [2]. As their name reflects (in Greek, “*atkis*” means ray and “*mykes*” means fungus), they share some morphological features with fungi [3]. They show filamentous growth, producing aerial or substrate mycelium. Actinomycetes are responsible for earthy smell of the soil [1]. They are ubiquitous in nature, found both in terrestrial and aquatic habitats [1, 4], including mangroves and sea sediments [5]. They belong to both mesophilic and thermophilic groups [6], which broaden the range of habitats inhabited by them. Actinomycetes are known to produce an extensive range of bioactive compounds including various enzymes having multiple biotechnological applications.

Lignocellulolytic enzymes, one of the potent enzymes produced by actinomycetes, can be exploited widely in various lignocelluloses based industries [7]. Lignocellulases are hydrolytic enzymes capable of degrading tough lignocellulose in the plant biomass and include cellulases, hemicellulases, and lignolytic enzymes [8]. Lignocellulose is the most abundant renewable biomass on earth [9]. It refers to the main constituents of the plant matter, that is, cellulose, hemicellulose, and lignin [10]. Hydrolysis of lignocellulosic biomass is accomplished by lignocellulolytic enzymes, which are used in diverse applications [11]. Cellulases are used in production of bioethanol and biomethane, in ligand binding studies [12], textile industry, pulp and paper making, detergents industry, animal feed and food, and so forth [13]. Hemicellulases are employed in biobleaching, deinking of paper waste, clarification of fruit juices, upgradation of feed, fodder and fibres, and saccharification of hemicelluloses to xylose sugars [14]. Applications of lignin-degrading enzymes involve pretreatment of recalcitrant lignocellulosic biomass for biofuel production, use in paper industry, textile industry, food industry, wastewater treatment, bioremediation, organic synthesis, and cosmetic and pharmaceutical industries [15].

Lignocellulolytic enzymes can be obtained from diverse types of microorganisms including bacteria and fungi [16]. Among bacteria actinomycetes are an attractive group, being tapped for production of lignocellulases [7, 17–20]. In this review, the diversity and applications of lignocellulolytic actinomycetes have been discussed along with description of their lignocellulases enzyme systems involved in biomass degradation.

## 2. Lignocellulose: Structure and Uses

**Figure 1:**
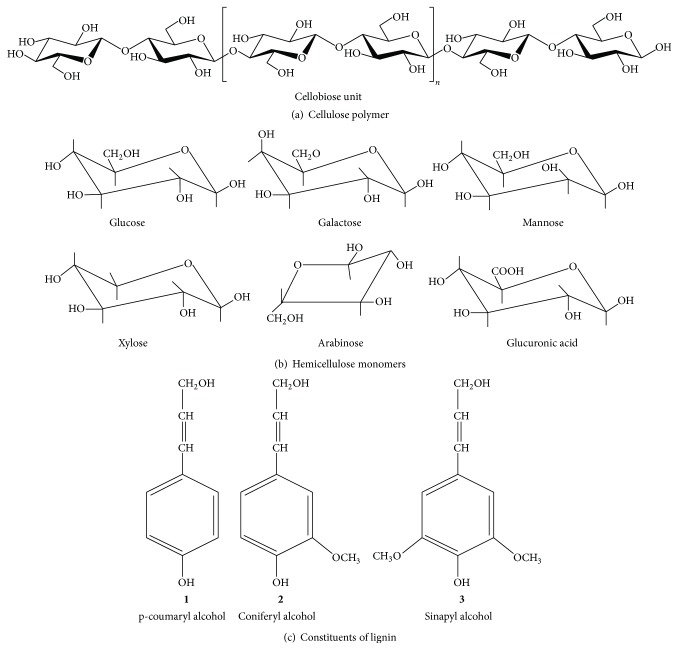
Chemical structure of lignocellulose.

Lignocellulose is comprised of three main components, that is, cellulose, hemicellulose, and lignin [21] (Figure 1). Cellulose is the high molecular weight linear polymer of D-glucopyranose units linked together by *β*-(l→4)-glycosidic bonds, with cellobiose dimer being the repeating unit. The cellulose chains are hydrogen bonded to each other, making a bundle of microfibrils, which further aggregate together to make cellulose fibrils [22]. The structure shows variations from amorphous to crystalline regions. The cellulose fibrils are packed in the cell wall in a matrix of hemicelluloses and lignin [23]. Hemicelluloses are linear or branched heteropolysaccharides composed of D-xylose, L-arabinose, D-galactose, D-glucose, or D-mannose sugars with or without different uronic acids and may include xylans, mannans, glucans, glucuronoxylans, arabinoxylans, glucomannans, galactomannans, galactoglucomannans, *β*-glucans, and xyloglucans [10, 24]. The branching and composition vary among different plant sources. Xylan, the polymer of xylose, is the most abundant hemicellulosic component [10]. Lignin is a very complex polyphenolic heteropolymer primarily made up of three phenyl propionic alcohol monomers, that is, p-coumaryl alcohol, coniferyl alcohol, and sinapyl alcohol [25].

Celluloses, hemicelluloses, and lignin are packed closely in a crisscross network and glued with the help of a variety of noncovalent and covalent linkages. Lignin acts as a cementing agent and provides structural support. It also provides impermeable barrier to the enzymes, making whole structure robust and resistant [22]. Cellulose is the most abundant polymer of plant cell walls (35–50%), followed with hemicellulose (20–35%) and lignin (10–25%) [10].

Cellulose is used for many purposes such as in manufacturing of paper and textile fabric, in production of biofuel (from fermentable sugar glucose), as inert packing and insulating material, and in food and drugs as base and stabilizer [26]. Hemicelluloses are utilized in a range of applications. Biodegradability and nontoxicity of hemicelluloses enable it to be used as dietary fibre [27] and as edible coating over foodstuffs for their stabilization. Mannans have cholesterol lowering properties [28]. Xyloglucans and *β*-glucans are used in ice-creams and other foods due to their gelling, stabilizing, and thickening effects. Adhesive properties are used in paper making. Hemicelluloses are rich source of xylose sugars, which are fermented to ethanol fuel [29]. Characteristics of lignin also make it an important component used in the production of a wide range of value added products [30]. Lignin can be used as a source of activated carbon and phenol useful in the synthesis of wide array of chemicals [31] and to produce biocomposites [32] or polymers [33] which can replace plastic based products either partially or completely. It can be used as binder in small scale processes and as filler in place of oil based carbon fibres. Biosorption properties of lignin [34] are also foreseen as a potent application. Furthermore, lignin is a rich source of energy [35]. Thus, lignocellulose is a repertoire of industrially useful components. But the structural characteristics of the cell wall or lignocellulose are such that the extraction of individual component to homogeneity and purity is a difficult task requiring specific chemical or biological procedures. Also many applications are based on degradation products of the cellulose, hemicellulose, or lignin making hydrolysis an essential requirement. Hydrolysis can be mediated both chemically and enzymatically. Chemical methods involve use of expensive chemicals, harsh conditions and have certain other limitations. Enzymatic methods are, therefore, preferred and lignocellulolytic enzymes play very important role in these applications. Presently biomass utilization is executed through biorefinery, which aims at significant utilization of all components generating least possible amount of waste. When one constituent of biomass is utilized, others are simultaneously used for production of valuable products. During biofuel production, sugars from hemicellulose and cellulose components are used in ethanol formation and lignin residue left is used for power generation or in other uses of lignin. Lignocellulases perform important functions in biorefinery processes also.

## 3. Lignocellulolytic Enzyme Systems in Actinomycetes

### 3.1. Cellulases

**Figure 2:**
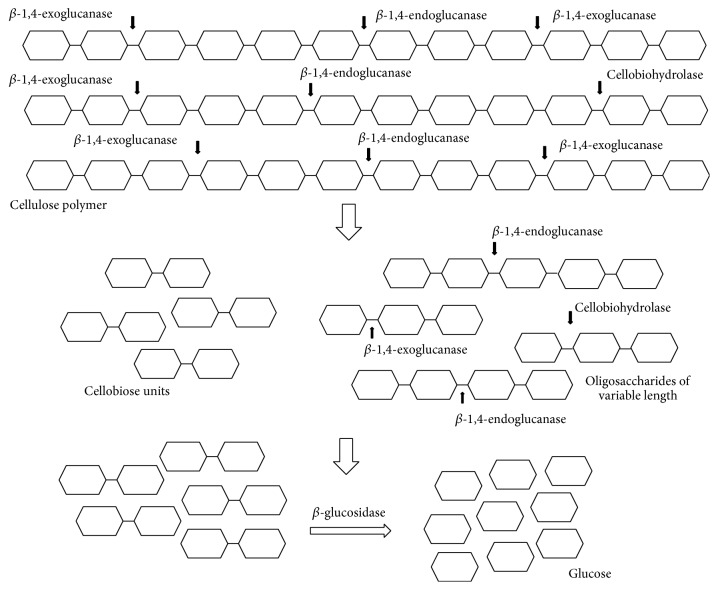
Scheme of cellulose hydrolysis.

Cellulolytic enzymes are a group of glycosyl hydrolases classified into different families depending on their sequence homologies. The mechanisms of action and substrate specificities vary among different cellulases, but they are generally divided into exoglucanases (EC 3.2.1.74), endoglucanases (EC 3.2.1.4), cellobiohydrolases (EC 3.2.1.91), and *β*-glucosidases (EC 3.2.1.21) [36, 37]. Exoglucanases act on reducing or nonreducing ends of cellulose chains releasing glucose units, whereas endoglucanases hydrolyse *β*-1,4-glycosidic bonds randomly inside the cellulose chains releasing dextrans of variable lengths [38]. Cellobiohydrolases cleave glycosidic bonds at nonreducing ends and release cellobiose units [39]. These enzymes are particularly important in hydrolysing crystalline cellulose because of their processivity [36]. *β*-glucosidases enzymes take part in hydrolysis of cellobiose units to monomeric glucose [38]. Complete hydrolysis of cellulose involves synergistic effect of all these enzymes, showing synergy between endoglucanases and exoglucanases (endo-exo synergy), exoglucanases acting on the reducing and nonreducing ends (exo-exo synergy), between cellobiohydrolases and *β*-glucosidases, and between catalytic and carbohydrate binding domains [39].  Figure 2 shows schematic presentation of enzymatic hydrolysis of cellulose polymer.

Microbial cellulase systems are either complexed or noncomplexed [39]. Complexed systems, known as cellulosomes, are characteristics of anaerobic bacteria, consisting of multienzyme complex protuberances from cell surface stabilized by dockerin and adhesion proteins. In aerobic bacteria, including most of the actinomycetes, cellulases are noncomplexed or free and are secreted extracellularly using specific secretion pathways.

Among cellulase producing actinomycetes,* Cellulomonas fimi*,* Microbispora bispora*, and* Thermobifida fusca* have been studied extensively [6, 39].* Thermobifida fusca* is a thermophilic, spore forming actinomycete [6]. The genome of* T. fusca* consists of 3.6 billion bp in a single circular chromosome, with 3117 coding sequences, and has 67.5% G+C content which stabilizes DNA in extreme temperature conditions [40]. The genome encodes for 36 glycoside hydrolases distributed in 22 GH (glycoside hydrolases) families [40]. Cellulase system of* T. fusca* is comprised of six extracellular cellulases (4 endocellulases and 2 exocellulases) and one intracellular *β*-glucosidase [6, 40–42]. Each enzyme has a separate catalytic and carbohydrate binding domain, both linked together with a linker peptide [36, 41]. Carbohydrate binding domains in all six cellulases belong to the same family, that is, 2CBD [41]. The catalytic domains, however, are different in all enzymes belonging to different families, with Cel5A and Cel5B from GH family 5, Cel6A and Cel6B from GH family 6, Cel9A and Cel9B from GH family 9, and Cel48A from family 48 [43]. Cel5A, Cel5B, Cel6A, and Cel9B are endocellulases and do not show processivity [36, 40, 41]. Cel6B and Cel48A are processive exocellulases which act at nonreducing and reducing ends, respectively. Cel9A is a novel processive cellulase with both exo- and endocellulase actions, starting exohydrolysis from the nonreducing end [36]. The position of CBD varies in different cellulases, which is N-terminus in Cel5A, Cel6B, and Cel48A and C-terminus in Cel6A, Cel9A, and Cel9B [41]. Sequence studies of catalytic domains have revealed less than 31% similarity between cellulases from taxonomically similar as well as dissimilar microbes, which indicates development of cellulase system as a result of horizontal gene transfer compared to the gene duplication [44]. The three-dimensional structure of Cel6A shows *α*-*β* barrel with a deep active site cleft formed by one shorter and one turned loop, consisting of four conserved Asp residues [42, 45]. Active site of GH48 is also in a cleft [42]. Structural characteristics of exocellulases allow them to bind processively, whereas open active sites of endocellulases enable them to bind cellulose internally at random sites [42]. Structural elucidation of Cel9A has shown that it gives activity between exo- and endocellulases because its weak binding domain 3c CBD is aligned with the active site in the catalytic domain, allowing processive hydrolysis by the enzyme [42].* T. fusca* also produces cellulose and chitin binding proteins, E7 and E8, the CBM33 proteins which improve cellulose hydrolysis mediated through exoglucanases [36, 40, 42]. Cellulases from* T. bifida* have also been found showing synergism with endocellulases and* Trichoderma reesei* CBHI [6].

*Cellulomonas fimi* is a facultative anaerobe, but it does not consist of cellulosomes of cellulolytic anaerobes; rather it produces free cellulases [46]. Similarly, facultatively anaerobic* Cellulomonas flavigena* also secrets free cellulases [46]. Both carry out efficient hydrolysis of celluloses and hemicelluloses. The cellulase enzyme systems in* Cellulomonas fimi* also consist of six cellulases [39], that is, three endocellulases (CenA, CenB, and CenD), two exocellulases (CbhA and CbhB), and a processive endocellulase, CenC [41, 46]. All these enzymes have activities similar to that in* T. bifida*, with same families of CBDs (2CBD) and catalytic domains, but different sequences [41, 47]. These cellulases are primarily secreted by sec dependent pathway [46] and, therefore, do not require intracellular folding or cofactors for their activity. In* C. fimi* ATCC 484 and* C. flavigena* ATCC 482, another enzyme GH94 (cellobiose phosphorylase) has also been discovered [46].* Microbispora bispora* also shows synthesis of six different cellulases, showing exo-exo and endo-exo synergism [6]. Genomic studies of* Streptomyces* sp. SirexAA-E (ActE), isolated from pine-boring woodwasp* Sirex noctilio*, have also shown genes for GH48 (CBH activity), GH74 (endocellulase), and CDB33 [48].* Streptomyces coelicolor* consisted of 221 carbohydrate active enzymes (CAZy) or 154 glycosyl hydrolases (GHs), encoded within 8.6 billion bp long genome [48].

The expression of cellulolytic genes in* T. bifida* is induced by cellobiose, whereas easily utilizable sugar glucose shows catabolite repression as in most of the other cellulolytic microbes [49]. The regulation of cellulolytic genes is mediated by the CelR repressor, which binds to a 9–14 bp palindrome (5′-TGGGAGCGCTCCCA-3′) in region 5′-upstream of the cellulase genes in the absence of cellobiose or cellulose [50]. During induction, cellulose is hydrolysed to cellobiose by constitutive cellulases. Cellobiose binds to CelR releasing it from the promoter, allowing transcription of the downstream genes [47]. In addition to the presence of glucose, cellulase genes expression is also regulated by cAMP levels.

The extracellular cellulases are secreted by actinomycetes using either one or both of the common bacterial systems for secretion of extracellular proteins, that is,* sec* general secretion system and* sec* independent twin-arginine translocation (TAT) systems. The general* Sec*retion route catalyses transmembrane translocation of proteins in their unfolded conformation, whereas* T*win-*a*rginine (TAT) system translocates secretory proteins in their native folded state. In* T. bifida* both of these systems were discovered, whereas* S. coelicolor* mainly utilizes TAT systems for protein export [40].

### 3.2. Hemicellulases

Hemicellulases are generally synthesised along with cellulases [36, 39]. Xylan and mannan are most abundant components of hemicelluloses. Complete hydrolysis of xylan involves an enzyme system consisting of endo-1,4-*β*-xylanases (EC 3.2.1.8), *β*-D-xylosidases (EC 3.2.1.37), *α*-L-arabinofuranosidases (EC 3.2.1.55), *α*-glucuronidases (EC 3.2.1.139), acetyl xylan esterases (EC 3.1.1.72), and ferulic/coumaric acid esterases (EC 3.1.1.73). Mannan is hydrolysed primarily by synergistic action of mannanases (EC 3.2.1.78), *β*-mannosidases (EC 3.2.1.25), and *α*-galactosidases (EC 3.2.1.22) [36, 51]. Mannanases hydrolyze *β*-1,4-glycosidic bonds internally, *β*-mannosidase cleave *β*-1,4 linked mannose from nonreducing ends, and *α*-galactosidase removes terminal D-galactosyl residues linked by *α*-1,6 linkages [52]. Degradation of mannan and xylan also enhances cellulose hydrolysis as they are known to inhibit cellulase activities [36].

Most of the hemicellulases belong to glycosyl hydrolases families; however, some enzymes involved in hemicellulose hydrolysis belong to glycosyltransferases (EC 2.4.1.x) [39]. Xylanases, hydrolysing internal *β*-1,4-glycosidic bonds, are classified into GH families 5, 7, 8, 10, 11, and 43. *β*-D-xylosidases hydrolyse xylose monomers from nonreducing ends of xylan oligosaccharides and belong to GH families 3, 39, 43, 52, and 54 [53]. Studies have indicated production of several xylanases by* T. bifida* and other actinomycetes.* T. bifida* has been found to be producing *β*-1,4-endoxylanases (xyl10A, xyl10B, and xil11A), xylosidases, *α*-L-arabinofuranosidases, xyloglucanases, *β*-1,3-glucanases (GH81), and *α*-N-arabinofuranosidases (xil43) [36, 40].* Cellulomonas fimi* synthesises extracellular endo- as well as exo-xylanases: xylan binding domain CBM4, *β*-mannanase, mannosidase, and xel74 (xyloglucan specific *β*-1,4-glucanase) [36, 46].* Cellulomonas flavigena* ATCC 482 are known to synthesize an unusual mixture of 19 endoxylanases, along with GH10, GH11, and GH30 xylanases; GH43 (*β*-xylosidase), GH51 *α*-arabinofuranosidase, and *α*-glucuronidase; GH26 and GH13 mannans; and GH16 and GH81 *β*-glucanase [46].* Streptomyces flavogriseus* has shown production of *β*-1,4-glucan glucanohydrolase [54]. Xylanase genes GH5 (*β*-mannosidase), GH10 (beta xylanase), GH11 (beta xylanase), CE4 (acetylxylan esterase) and GH6 (CBH), and GH9 (CBH) have also been found in* Streptomyces* sp. SirexAA-E (ActE) [48].

### 3.3. Lignolytic Enzymes

Lignin degradation is mediated by a complex of enzymes containing three principal enzymes laccases (EC 1.10.3.2), manganese peroxidases (MnP, EC 1.11.1.13), and lignin peroxidases (LiP, EC 1.11.1.14) [55, 56]. Laccases are the oxidoreductases which degrade polyphenol, the principal recalcitrant component in the lignocellulose [15, 57]. They are extracellular inducible enzymes which employ simple oxygen as an oxidizing agent as well as cofactor. They are multicopper oxidases having four copper atoms in their active sites, taking part in oxygen reduction [58]. Low substrate specificity of laccases enables them to degrade wide variety of compounds. Manganese and lignin peroxidases are together known as heme peroxidases containing protoporphyrin IX as a prosthetic group. Lignin peroxidases can specifically degrade high redox potential compounds and are known to oxidize phenolic as well as nonphenolic aromatic rings, which make up around 90% of the lignin polymer. They require H_2_O_2_ for their activity. Veratryl alcohol is an attractive substrate for LiP, which oxidises other substrates by acting as the redox mediator for indirect oxidation. Manganese peroxidases are low redox potential heme peroxidases requiring H_2_O_2_ for their activity. They can be manganese dependent or versatile peroxidases [56, 58].

Laccases or Laccase-like multicopper oxidases containing (LMCO) four copper atoms are classified in types 1, 2, and 3 [57, 59]. The four copper atoms are distributed in three domains in most of the bacterial and fungal laccases [60]. Structural studies in several actinomycetes, however, have revealed presence of two Cu-binding domains, rather than three [61, 62]. The two-domain structure has been named as small laccase or small LMCO [59, 61]. LMCOs in* Streptomyces griseus*,* Streptomyces cyaneus*,* Streptomyces coelicolor*,* Streptomyces ipomoea*,* Streptomyces sviceus*,* Streptomyces* sp., and* Thermobifida fusca* are active as dimers or trimmers [61, 63, 64].

## 4. Genetic Engineering

The genes from several lignocellulolytic actinomycetes have been successfully cloned to show heterologous expression in different microbes. GH1 and GH3 enzymes of* C. fimi* ATCC 484 expressed in* E. coli* have shown efficient hydrolysis of celluloses and xylanases [65]. CelStrep gene from cellulolytic* Streptomyces* sp. G12 cloned and expressed in* E. coli* was found to belong to GH12 family and catalysed hydrolysis of carboxymethylcellulose following a Michaelis-Menten kinetics with a *K*_*m*_ of 9.13 mg/mL and a *V*_max_ of 3469 *μ*M min^−1^ [66].* Streptomyces reticuli* consists of Cel1 gene encoding for avicelase enzyme which alone can hydrolyse crystalline cellulose effectively [67]. When this gene was cloned and expressed in* E. coli*,* Bacillus subtilis*, and* Streptomyces* spp., enzyme was produced but in lower amounts probably due to the absence of genes encoding for essential regulatory factors [68]. From xylanolytic* Actinomadura* sp strain FC7 two genes, xylI and xylII, have been cloned, expressed, and well characterized in* Streptomyces lividans* [69]. Xylanase gene xylBS27 belonging to GH11 from* Streptomyces* sp. S27 has been successfully cloned and expressed in* Pichia pastoris*, hydrolysing xylan to xylobiose [70]. Similarly, expression of laccase gene from* Streptomyces coelicolor* (SLAC) in* Streptomyces lividans* produced large amount of high purity laccase (350 mg L^−1^) [71]. The gene for a thermostable laccase from* Streptomyces lavendulae* REN-7 was successfully cloned and expressed in* E. coli* [72]. Cloning of a lignin peroxidase from* Streptomyces viridosporus* T7A into* Streptomyces lividans* TK64 has resulted in better lignocellulose degradation by genetically engineered* S. lividans* compared to* S. lividans* TK64 [73]. Thus, genetic engineering techniques can be and are being used for constructing industrially valuable strains with potent applications based on actinomycetes lignocellulolytic enzymes.

## 5. Diversity of Lignocellulolytic Actinomycetes

### 5.1. Cellulolytic Actinomycetes

Cellulolytic potential of actinomycetes has been explored since inspection of other microorganisms for cellulase production. Various research studies support high cellulose degradation potential of microbes from actinomycetales.  Table 1 represents the diversity of actinomycetes producing cellulase enzymes.

### 5.2. Hemicellulolytic Actinomycetes

Diverse types of actinomycetes belonging to wide range of habitats and active in different environmental conditions are known to produce hemicellulolytic enzymes.* Streptomyces* have been found to be the most abundant hemicellulases producer among actinomycetes. In a study by Boroujeni et al. [74], all of the isolated hemicellulolytic actinomycetes were found to belong to* Streptomyces* genus. Xylanase has been successfully purified from* Streptomyces* sp. E-86 and characterized for its xylanolytic activity [75]. Optimization studies were carried out for endoxylanase production by* Streptomyces* sp. F2621 isolated from Turkey [76]. *β*-xylosidase activity of* Streptomyces* has been used in saccharification of ball milled wheat straw [77]. A thermostable xylanase was obtained from* Streptomyces* sp. QG-11-3, which has shown biobleaching effects in eucalyptus kraft pulp [78]. Xylanase has also been produced from other strains of* Streptomyces* sp. such as* Streptomyces* sp. strain C1-3 [79],* Streptomyces* sp. CD3 [80],* Streptomyces* sp. 7b [81],* Streptomyces* sp PC22,* Streptomyces* sp 234P-16I, SWU-10,* Streptomyces* sp. MDS [82], and* Streptomyces* sp. [83]. In* Streptomyces rochei* and* Streptomyces chromofuscus*, xylanase production has been achieved using treated Papyrus and cotton stalk pulp. The obtained xylanase when used for studying bleaching effects has shown enhanced brightness in the presence of EDTA [84]. Xylanases have also been obtained from* Streptomyces albus* and* Streptomyces hygroscopicus* and have shown successful production of biogas using oil cake and straw waste [85].

In a study by Ninawe et al. [86], three* Streptomyces* isolates, that is,* Streptomyces cyaneus*,* S. tendae*, and* S. caelestis*, were found to be xylanolytic and the enzyme from* Streptomyces cyaneus* was successfully purified followed with its characterization [87].* Streptomyces thermoviolaceus* OPC-520 exhibited production of acetyl xylan esterases and *α*-L-arabinofuranosidases enzymes [88]. Extracellular xylanase production has also been observed in* Streptomyces aureofaciens* [89] and in* Streptomyces coelicolor* grown on different agricultural wastes such as sugarcane bagasse, pineapple, orange, and pomegranate peels [90]. Xylanases from* Streptomyces albus* and* Streptomyces chromofuscus* have indicated positive bleaching effects in rice straw pulp [91]. Bhosale et al. [92] have shown production of 326 IU/mL of xylanases from* Streptomyces rameus* using sugarcane bagasse along with peptone and dextrose [92]. Studies have indicated production of cellulase free xylanases from* Streptomyces roseiscleroticus* [93, 94] and* Saccharomonospora viridis* [95]. Improvement in xylanase production has been documented in* Streptomyces pseudogriseolus* subjected to UV mutagenesis [96]. Xylanase production has also been seen in* Thermomonospora fusca* [77, 97].* Thermomonospora curvata*,* Thermomonospora alba*,* Micromonospora*,* Microbispora bispora*,* Nocardia*,* Saccharomonospora viridis*, and* Thermoactinomyces* have shown production of *β*-xylosidases, acetylesterases, and arabinofuranosidases [77]. Extracellular xylanases have been partially purified and characterized in* Microbispora siamensis* in a study by Boondaeng et al. [98]. Xylanase production has also been observed in* Microtetraspora flexuosa* [99],* Streptomyces chattanoogensis* UAH 23 [100],* Streptomyces chattanoogensis* CECT 3336 [101],* Streptomyces violaceoruber* [102],* Thermoactinomyces thalophilus* [103],* Thermomonospora* sp. [104],* Streptomyces thermocyanaeviolaceus* [105], and* Streptomyces lividans* [106]. The enzyme from* Streptomyces lividans* was purified and characterized by different researchers [107, 108].* Microbispora* sp. has been found to be producing hemicellulolytic mannanase enzyme [109], whereas other studies have indicated production of *β*-xylosidases by* Streptomyces albogriseolus*,* S. nitrosporeus*, and* Micromonospora melanosporea* [110].

### 5.3. Lignolytic Actinomycetes

Lignolytic activity is exhibited by diverse range of actinomycetes, which play important role in biodegradation processes in the environment. Search is in progress for more actinomycetes with high lignolytic potential, using advanced techniques combined with conventional methods. A study by Fernandes et al. [111] have used specifically designed primers for detection of laccase-like genes within actinomycetes and has identified gene fragments undetectable by known primers, which corresponded to superfamilies I and K based on laccase and multicopper oxidase engineering database. Arias et al. [112] have shown production of laccase by* Streptomyces cyaneus* CECT 3335 using soya flour. Laccase was purified and characterized and was found to show increase in brightness of eucalyptus kraft pulp in biobleaching studies. The enzyme was able to oxidize veratryl alcohol suggesting potential of the strain in industrial applications. Veratryl alcohol oxidation and other lignolytic activities have also been demonstrated in* Streptomyces viridosporus* [113, 114].

*Streptomyces* sp. strain EC-22, strain EC1,* Streptomyces badius*,* Streptomyces cyaneus* MT813,* Thermomonospora fusca*,* Thermomonospora chromogena*,* Thermomonospora mesophila*,* Amncolata autotirophica*, and* Micromonospora* sp. have shown significant activities against lignin related compounds [115, 116]. Pasti et al. [117] have shown lignolytic activity in* Streptomyces chromofuscus*,* Streptomyces diastaticus*, and* Streptomyces rochei*. Several actinomycetes such as* Streptomyces coelicolor*,* Streptomyces griseus*, and* Nocardia* and several strains of* Streptomvces* sp. isolated from termite* Amitermes hastatus* have indicated production of laccases, lignin peroxidases, or manganese peroxidases enzymes by them [118]. Laccase and lignin peroxidase activities have also been observed in* Streptomyces cinnamomeus* [119]. Laccase enzyme studies were carried out including their structural elucidation in* Streptomyces lavendulae*,* Streptomyces psammoticus*,* Streptomyces ipomoeae*, and* Streptomyces sviceus* [61]. In a study by Escudero et al. [120],* Tsukamurella* and* Cellulosimicrobium* actinomycetes showed ABTS (2,2′-azino-bis-3-ethylbenzothiazoline-6-sulphonic acid) oxidation rate of 108 U/L and 0.56 U/L, respectively [120]. Submerged fermentation in marine actinomycete* Streptomyces lydicus*, isolated from Egypt Red Sea, grown in medium supplemented with peanut shell, produced 1.625 U/mL laccase under optimized conditions [121]. Thermoalkali stable laccase from* Thermobifida fusca* has shown oxidation of several dye intermediates including 2,6-dimethylphenylalanine and p-aminophenol [64].* Streptomyces sviceus* was also found to be showing lignolytic activity [63].* Streptomyces psammoticus* has shown enhanced production of laccase under solid state fermentation conditions in the presence of pyrogallol inducer, which was taken to the level of scale-up studies using a packed bed bioreactor [122]. Study by Niladevi and Prema [123] has shown production of all three enzymes, that is, laccase, manganese peroxidase, and lignin peroxidase, by* Streptomyces psammoticus*. Actinomycete* Rhodococcus ruber* has shown oxidation and degradation of polyethylene as a result of laccase production by it [124]. A study by Aoyama et al. [125] demonstrated laccase production by* Streptomyces atratus*. In search of the genes involved in lignocellulose degradation during composting of agricultural wastes, two-domain laccase-like multicopper oxidase genes were identified in* Streptomyces violaceusniger* [62].* Rhodococcus jostii* was found to produce lignin peroxidases capable of modifying lignin [126]. Several other studies have shown lignin degradation ability in many other actinomycetes including* Streptomyces flavovirens* [127],* Streptomyces setoni* [128],* Actinomadura* spp. [55], and* Streptomyces thermoviolaceus* [129].

## 6. Future Prospects

Owing to the abundance and renewability of lignocellulosic biomass, it is considered as most appropriate and economical feedstock for production of various industrially useful products. Lignocellulases enzymes are, therefore, critical in processes associated with bioconversion of lignocelluloses. Presently most of the commercially exploited lignocellulases rely on fungal or bacterial microorganisms. Actinomycetes are relatively less explored for their biomass hydrolysis potential. The studies can be elaborated in search of new actinomycetes producing lignocellulose degrading enzyme systems. Different feedstock shows variation in their chemical composition. The production of enzymes needs to be optimized for different biomass. The production of lignocellulases from all microbial sources is still quite expensive. Efforts can be made for reducing the cost of production of these enzymes using high potency actinomycete enzyme systems with broader range of tolerance and active at diverse environmental conditions. Genetic engineering techniques can be used to construct enzyme systems with desirable characteristics. Also the studies can be expanded gradually to scale up to the industrial levels for their subsequent adoption in commercial processes.

## 7. Conclusion

Actinomycetes are an important source of lignocellulose hydrolysing enzymes. They constitute considerable proportion of the soil or aquatic microflora responsible for biomass degradation in nature. The research studies on search of lignocellulose hydrolysing actinomycetes revealed the abundance and diversity of these microbes in different ecological niches. The genetic and protein studies on their hydrolytic enzymes lead to the elucidation of structural and mechanism details of enzymes and their relatedness with other known lignocellulose producers and their enzyme systems. Relatively scanty information is available on lignocellulolytic actinomycetes. The research studies, therefore, need to be elaborated in view of utilization of lignocellulolytic potential of actinomycetes applicable in different industrial sectors.

## Figures and Tables

**Table 1:** Cellulase producing actinomycetes.

Actinomycete isolate	Cellulolytic enzyme	Observed results	Reference
*Streptomyces *sp.	*β*-glucosidases	Saccharification of rice straw	[77]
	Extracellular cellulases	Zone of hydrolysis in plate assay method	[109, 130]
	Endoglucanases	Enzyme production	[131]
	Cellulases	Aid in composting	[132]
*Streptomyces *sp.strain AT7	Carboxymethylcellulose (Cx) and Avicelase (C1) enzyme	Production of enzymes	[133]
*Streptomyces *sp.strains M7a and M7b, F2621, LIPIMC-A-194, LIPIMC-A-251, and LIPIMC-A-278	Endoglucanases/CMCase (carboxymethylcellulose)	CMCase production	[76, 134, 135]
*Streptomyces *sp.T3-1		Scale-up of enzyme production	[136]
*Streptomyces *sp.S7		Enzyme production using fruit waste	[137]

*Streptomyces albogriseolus,* *Streptomyces nitrosporeus*	*β*-glucosidase, endoglucanase, and avicelase	Optimization studies for production of cellulases	[110]

*Streptomyces lividans*	Cellulases	Enzyme characterization Enzyme production optimization	[106] [138]

*Streptomyces flavogriseus* * Streptomyces nitrosporus*	Cellulases	Optimization of enzyme production	[138]

*Streptomyces albaduncus*	Exoglucanase, *β*-glucosidase, and endoglucanase	Catabolite repression studies Characterization of enzyme	[139] [140]

*Streptomyces reticuli*	Avicelase	Gene expression studies	[68]

*Streptomyces cellulolyticus*	Extracellular cellulases	Cellulose decomposition	[141]

*Streptomyces drozdowiczii*	Endoglucanase	Successful application in detergent and textile processing	[142]

*Streptomyces malachitofuscus*, *Streptomyces glomeratus*, and *Streptomyces stramineus*	Extracellular cellulases	Zone of hydrolysis in plate assay method	[109]

*Streptomyces gancidicus*	Extracellular cellulase, CMCase/endoglucanase	Zone of hydrolysis in plate assay method	[109, 143]

*Streptomyces actuosus*	Endoglucanase	Optimization of CMCase production	[144]

*Streptomyces bobili *LIPIMC-A-283, *Streptomyces olivochromogenes* LIPIMC-A-247	CMCase/endoglucanase	CMCase production	[135]

*Streptomyces globosus*, *Streptomyces alanosinicus*,	CMCase/endoglucanase	Zone of hydrolysis in plate assay method	* * [143]
*Streptomyces ruber*		Optimization studies	

*Streptomyces viridobrunneus* SCPE-09	Endoglucanase	Enzyme production from agroindustrial residues	[145]

*Streptomyces viridiochromogenes*	Avicelase, CMCase, and total cellulase	Saccharification of rice straw and ethanol production	[146]

*Streptomyces albospinus*	Extracellular cellulases	Degradation of cellulosic materials	[147]

*Streptomyces griseorubens*	Cellulases	Enzyme production optimization	[148]

*Streptomyces matensis*	Endoglucanase and exoglucanase	Enzyme production optimization	[149]

*Streptomyces longispororuber*	Carboxymethylcellulose	Production and purification of enzyme	[150]

*Cellulomonas fmi*	Cellulase	Activity observed,	[151] [152]
	FPase (total cellulases)	saccharification of rice straw and ethanol production, and optimization of enzyme production using wheat straw	[153]

*Cellulomonas fimi*			
*Cellulomonas *sp.			
*Cellulomonas *sp. (strains ATCC21399 and JHHY35)	CMCase, FPase	CMC depolymerisation and filter paper disintegration	[154]
*Cellulomonas cartae*			
*Cellulomonas cellulase*			
*Cellulomonas cellulase *			
*Cellulomonas flavigena*			
*Cellulomonas subalbus*		Degradation of flax, sisal, and cotton fibres	
*Cellulomonas uda*			
*Cellulomonas biazotea*			
*Cellulomonas gelida*			[155]

*Cellulomonas biazotea*	FPase, endo-*β*-glucanase and *β*-glucosidase endo- and exoglucanases, and cellobiases	Enzyme production using cellulosic substrates Cellulosic biohydrogen production	[156] [157]

*Cellulomonas cellulans*	CMCase	Degradation of flax, sisal, and cotton fibres	[155]
*Cellulomonas cellulans *NRRL B 4567		Study of substrate inhibition kinetics	[158]
*Cellulomonas *	Endoglucanase/CMCase	Enzyme production using cassava bagasse	[159]
*cellulans*		Isolation from silver fish and cellulase characterization	[160]

*Intrasporangium, Saccharopolyspora,* *Streptosporangium, Rhodococcus, Saccharomonospora, *and* Nocardia* *Micromonospora*	Extracellular cellulase	Zone of hydrolysis in plate assay method	[130]

*Micromonospora*	*β*-glucosidase	Saccharification of rice straw	[77]
*Micromonospora chalcea*	*β*-glucosidase and CMCase	Demonstration of activity	[161]
		Enzyme production	[162]

*Microbispora bispora* *Microbispora *sp. *Micromonospora melanosporea* *Microbispora bispora*	*β*-glucosidase Cellulase *β*-glucosidase, endoglucanase & Avicelase Endoglucanase/CMCase	Saccharification of rice straw Help in composting Optimization of cellulase production Enzyme production	[77] [132] [110] [163]

*Thermomonospora *sp.	*β*-glucosidase	Saccharification of rice straw	[77]
	CMCase	Enzyme production, purification, and characterization	[164]
*Thermomonospora curvata*	Extracellular cellulase	Production and partial purification of enzyme	[165]

*Actinoplanes minutisporangius* LIPIMC-A-269 and 279	CMCase/endoglucanase	CMCase production	[137]

*Streptoverticillium morookaense*, *Nocardiopsis aegyptia*	CMCase/endoglucanase	Zone of hydrolysis in plate assay method	[143]

*Pseudonocardia thermophila*	*β*-glucosidase and CMCase	Activities demonstrated	[161]

*Actinopolyspora halophila*	CMCase and *β*-glucosidase	Detection of activity	[166]

*Thermoactinomyces *sp.	Endoglucanase, *β*-glucosidase, and avicelase	Degradation of microcrystalline cellulose	[167]

*Thermoactinomyces *sp.strain TA3	Extracellular cellulase	High efficiency and bioactivity observed during compositing	[168]
